# 2-(1*H*-Benzotriazol-1-yl)-1-(4-bromo­benzo­yl)ethyl 2-chloro­benzoate

**DOI:** 10.1107/S1600536809002712

**Published:** 2009-01-31

**Authors:** Kong-Cheng Hu, Guang-Jiu Li

**Affiliations:** a College of Life Science and Pharmaceutical Engineering, Nanjing University of Technology, 210009 Nanjing, Jiangsu, People’s Republic of China

## Abstract

In the title compound, C_22_H_15_BrClN_3_O_3_, the benzotriazole ring system makes dihedral angles of 2.43 (1) and 71.51 (1)° with the bromo­phenyl and chloro­phenyl rings, respectively; the angle between the latter two rings is 69.26 (1)°. In the crystal structure, mol­ecules are linked into chains by inter­molecular C—H⋯O hydrogen bonds. The crystal packing is further stabilized by π–π (with a centroid-centroid distance of 3.764 Å) and C—H⋯π inter­actions.

## Related literature

For the crystal structure of a related compound, see: Zeng *et al.* (2007 ▶). For standard bond-length data, see: Allen *et al.* (1987 ▶).
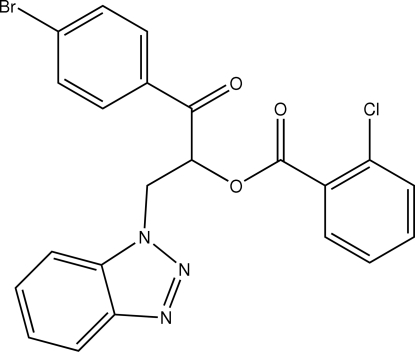


## Experimental

### 

#### Crystal data

C_22_H_15_BrClN_3_O_3_
*M* *_r_* = 484.72
Monoclinic, 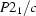

*a* = 6.2613 (7) Å
*b* = 36.688 (4) Å
*c* = 8.7919 (9) Å
β = 90.878 (2)°
*V* = 2019.4 (4) Å^3^
*Z* = 4
Mo *K*α radiation
μ = 2.20 mm^−1^
*T* = 293 (2) K
0.36 × 0.21 × 0.09 mm

#### Data collection

Siemens SMART 1000 CCD area-detector diffractometer
Absorption correction: multi-scan (*SADABS*; Sheldrick, 1996 ▶) *T*_min_ = 0.505, *T*_max_ = 0.827
11039 measured reflections
3978 independent reflections
2968 reflections with *I* > 2σ(*I*)
*R*_int_ = 0.025

#### Refinement

*R*[*F* ^2^ > 2σ(*F* ^2^)] = 0.041
*wR*(*F* ^2^) = 0.103
*S* = 1.04
3978 reflections
271 parameters
H-atom parameters constrained
Δρ_max_ = 0.48 e Å^−3^
Δρ_min_ = −0.44 e Å^−3^

### 

Data collection: *SMART* (Siemens, 1996 ▶); cell refinement: *SAINT* (Siemens, 1996 ▶); data reduction: *SAINT*; program(s) used to solve structure: *SHELXS97* (Sheldrick, 2008 ▶); program(s) used to refine structure: *SHELXL97* (Sheldrick, 2008 ▶); molecular graphics: *SHELXTL* (Sheldrick, 2008 ▶); software used to prepare material for publication: *SHELXTL*, *PARST* (Nardelli, 1995 ▶) and *PLATON* (Spek, 2003 ▶).

## Supplementary Material

Crystal structure: contains datablocks global, I. DOI: 10.1107/S1600536809002712/wn2306sup1.cif

Structure factors: contains datablocks I. DOI: 10.1107/S1600536809002712/wn2306Isup2.hkl

Additional supplementary materials:  crystallographic information; 3D view; checkCIF report

## Figures and Tables

**Table 1:** Hydrogen-bond geometry (Å, °) *Cg*1 is the centroid of the N1–N3/C17/C18 triazole ring.

*D*—H⋯*A*	*D*—H	H⋯*A*	*D*⋯*A*	*D*—H⋯*A*
C16—H16*A*⋯*Cg*1^i^	0.93	2.87	3.707	150
C21—H21*A*⋯O3^ii^	0.93	2.47	3.101 (4)	126

Symmetry codes: (i) 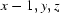; (ii) 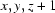.

**Fig. 1.**
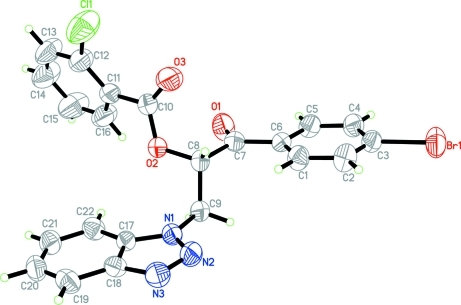
The molecular structure of the title compound, showing 50% probability displacement ellipsoids and the atom numbering scheme. Hydrogen atoms are represented by spheres of arbitrary radius.

**Fig. 2.**
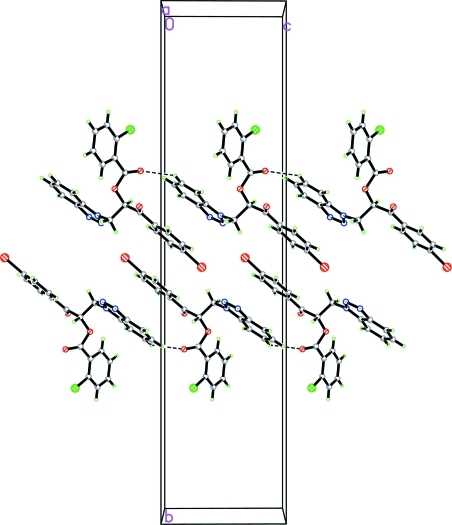
A packing diagram, viewed down the a axis. Hydrogen bonds are indicated by dashed lines.

## supplementary crystallographic information

### Comment

The crystal structure of 2-(benzotriazol-1-yl)-1-(4-methylbenzoyl)ethyl 4-ethylbenzoate has been reported by Zeng *et al.* (2007). In a search for new benzotriazole derivatives with higher bioactivity, the title compound was synthesized and its structure is reported here.

In the title molecule (Fig. 1), all bond lengths (Allen *et al.*, 1987) and angles are within normal ranges. The benzotriazole system is almost planar,  with a dihedral angle of 0.89 (1)° between the triazole ring (N1–N3/C17/C18) and the benzene ring A (C17–C22). The benzotriazole mean plane makes dihedral angles of 2.43 (1)° and 71.51 (1)° with benzene rings B (C1–C6) and C (C11–C16), respectively, indicating the non-planarity of the whole molecule. The dihedral angle between rings B and C is 69.26 (1)°.

In the crystal structure (Fig. 2), molecules are linked into chains along the *c* axis by intermolecular C21—H21A···O3 hydrogen bonds (Table 1). The distance of 3.764 Å between the centroids of rings A and B related by the symmetry code (*x*, *y*, -1 + *z*) suggests a possible π···π interaction. The crystal packing is further stabilized by C—H···π interactions (Table 1).

### Experimental

The title compound was prepared according to the literature method of Zeng *et al.* (2007). Single crystals suitable for X-ray diffraction were obtained by slow evaporation of an ethyl acetate solution at room temperature over a period of six days.

### Refinement

All H atoms were located in difference Fourier maps and constrained to ride on their parent atoms, with C(aromatic)—H = 0.93 Å, C(methylene)—H = 0.97 Å and C(methine)—H = 0.98 Å; *U*_iso_(H) = 1.2 *U*_eq_(C).

### Crystal data

**Table:**

C_22_H_15_BrClN_3_O_3_	*F*(000) = 976
*M**_r_* = 484.72	*D*_x_ = 1.594 Mg m^−^^3^
Monoclinic, *P*2_1_/*c*	Mo *K*α radiation, λ = 0.71073 Å
*a* = 6.2613 (7) Å	Cell parameters from 3180 reflections
*b* = 36.688 (4) Å	θ = 2.2–22.9°
*c* = 8.7919 (9) Å	µ = 2.20 mm^−^^1^
β = 90.878 (2)°	*T* = 293 K
*V* = 2019.4 (4) Å^3^	Plate, colourless
*Z* = 4	0.36 × 0.21 × 0.09 mm

### Data collection

**Table:**

Siemens SMART 1000 CCD area-detector diffractometer	3978 independent reflections
Radiation source: fine-focus sealed tube	2968 reflections with *I* > 2σ(*I*)
graphite	*R*_int_ = 0.025
Detector resolution: 8.33 pixels mm^-1^	θ_max_ = 26.1°, θ_min_ = 2.2°
ω scans	*h* = −7→7
Absorption correction: multi-scan (*SADABS*; Sheldrick, 1996)	*k* = −44→45
*T*_min_ = 0.505, *T*_max_ = 0.827	*l* = −10→8
11039 measured reflections	

### Refinement

**Table:**

Refinement on *F*^2^	Primary atom site location: structure-invariant direct methods
Least-squares matrix: full	Secondary atom site location: difference Fourier map
*R*[*F*^2^ > 2σ(*F*^2^)] = 0.041	Hydrogen site location: inferred from neighbouring sites
*wR*(*F*^2^) = 0.103	H-atom parameters constrained
*S* = 1.04	*w* = 1/[σ^2^(*F*_o_^2^) + (0.0492*P*)^2^ + 0.6802*P*] where *P* = (*F*_o_^2^ + 2*F*_c_^2^)/3
3978 reflections	(Δ/σ)_max_ < 0.001
271 parameters	Δρ_max_ = 0.48 e Å^−^^3^
0 restraints	Δρ_min_ = −0.43 e Å^−^^3^

### Special details

**Table:**

Geometry. All e.s.d.'s (except the e.s.d. in the dihedral angle between two l.s. planes) are estimated using the full covariance matrix. The cell e.s.d.'s are taken into account individually in the estimation of e.s.d.'s in distances, angles and torsion angles; correlations between e.s.d.'s in cell parameters are only used when they are defined by crystal symmetry. An approximate (isotropic) treatment of cell e.s.d.'s is used for estimating e.s.d.'s involving l.s. planes.
Refinement. Refinement of *F*^2^ against ALL reflections. The weighted *R*-factor *wR* and goodness of fit *S* are based on *F*^2^, conventional *R*-factors *R* are based on *F*, with *F* set to zero for negative *F*^2^. The threshold expression of *F*^2^ > σ(*F*^2^) is used only for calculating *R*-factors(gt) *etc*. and is not relevant to the choice of reflections for refinement. *R*-factors based on *F*^2^ are statistically about twice as large as those based on *F*, and *R*- factors based on ALL data will be even larger.

### Fractional atomic coordinates and isotropic or equivalent isotropic displacement parameters (Å^2^)

**Table:**

	*x*	*y*	*z*	*U*_iso_*/*U*_eq_	
Br1	0.29655 (6)	0.490655 (9)	0.67871 (4)	0.06861 (15)	
Cl1	0.06423 (18)	0.75440 (3)	1.24666 (14)	0.0966 (4)	
O2	0.0115 (3)	0.64041 (5)	1.3681 (2)	0.0511 (5)	
N1	0.3152 (3)	0.59119 (6)	1.5238 (2)	0.0427 (5)	
C3	0.1975 (5)	0.52390 (8)	0.8274 (3)	0.0463 (7)	
C18	0.5177 (4)	0.61307 (7)	1.7051 (3)	0.0413 (6)	
N2	0.5149 (4)	0.57773 (6)	1.5071 (3)	0.0518 (6)	
O1	−0.2188 (3)	0.60500 (7)	1.1628 (2)	0.0696 (7)	
C17	0.3102 (4)	0.61371 (7)	1.6470 (3)	0.0380 (6)	
C5	−0.0818 (5)	0.55733 (8)	0.9389 (3)	0.0501 (7)	
H5A	−0.2247	0.5642	0.9399	0.060*	
C6	0.0564 (4)	0.57085 (7)	1.0497 (3)	0.0406 (6)	
C21	0.2051 (5)	0.65309 (8)	1.8413 (3)	0.0524 (7)	
H21A	0.1020	0.6669	1.8898	0.063*	
C10	−0.0114 (5)	0.67132 (8)	1.2854 (3)	0.0567 (8)	
C9	0.1469 (5)	0.58149 (7)	1.4157 (3)	0.0458 (7)	
H9A	0.0144	0.5782	1.4698	0.055*	
H9B	0.1823	0.5585	1.3681	0.055*	
O3	0.0745 (5)	0.67563 (7)	1.1669 (3)	0.0951 (9)	
N3	0.6384 (4)	0.59032 (7)	1.6152 (3)	0.0523 (6)	
C20	0.4137 (5)	0.65259 (8)	1.9019 (3)	0.0524 (7)	
H20A	0.4446	0.6659	1.9895	0.063*	
C19	0.5718 (5)	0.63304 (8)	1.8356 (3)	0.0510 (7)	
H19A	0.7102	0.6330	1.8755	0.061*	
C22	0.1482 (5)	0.63403 (8)	1.7136 (3)	0.0486 (7)	
H22A	0.0099	0.6346	1.6736	0.058*	
C2	0.3391 (5)	0.53722 (8)	0.9333 (4)	0.0550 (8)	
H2B	0.4818	0.5303	0.9306	0.066*	
C8	0.1142 (5)	0.61048 (7)	1.2923 (3)	0.0472 (7)	
H8A	0.2516	0.6181	1.2510	0.057*	
C1	0.2703 (4)	0.56101 (8)	1.0444 (3)	0.0491 (7)	
H1A	0.3671	0.5704	1.1154	0.059*	
C4	−0.0131 (5)	0.53398 (8)	0.8277 (3)	0.0527 (8)	
H4A	−0.1079	0.5252	0.7540	0.063*	
C7	−0.0336 (5)	0.59603 (8)	1.1656 (3)	0.0472 (7)	
C16	−0.3280 (6)	0.68352 (9)	1.4437 (4)	0.0716 (10)	
H16A	−0.3416	0.6585	1.4564	0.086*	
C11	−0.1591 (5)	0.69699 (8)	1.3596 (3)	0.0524 (7)	
C13	−0.2875 (6)	0.75732 (9)	1.4121 (5)	0.0779 (11)	
H13A	−0.2728	0.7824	1.4022	0.093*	
C14	−0.4524 (6)	0.74321 (10)	1.4933 (5)	0.0866 (12)	
H14A	−0.5498	0.7588	1.5385	0.104*	
C15	−0.4754 (7)	0.70624 (11)	1.5085 (5)	0.0896 (13)	
H15A	−0.5894	0.6967	1.5621	0.108*	
C12	−0.1430 (5)	0.73429 (8)	1.3451 (4)	0.0599 (8)	

### Atomic displacement parameters (Å^2^)

**Table:**

	*U*^11^	*U*^22^	*U*^33^	*U*^12^	*U*^13^	*U*^23^
Br1	0.0825 (3)	0.0610 (2)	0.0628 (2)	−0.00151 (17)	0.01525 (18)	−0.02185 (16)
Cl1	0.0949 (7)	0.0628 (6)	0.1336 (10)	−0.0065 (5)	0.0472 (7)	0.0038 (6)
O2	0.0750 (14)	0.0417 (11)	0.0368 (10)	0.0131 (9)	0.0022 (9)	−0.0028 (8)
N1	0.0460 (13)	0.0434 (12)	0.0384 (12)	0.0057 (10)	−0.0040 (10)	−0.0041 (10)
C3	0.0568 (19)	0.0380 (14)	0.0443 (16)	−0.0017 (13)	0.0071 (13)	−0.0027 (12)
C18	0.0418 (15)	0.0445 (15)	0.0375 (14)	0.0020 (12)	0.0005 (12)	0.0035 (11)
N2	0.0537 (15)	0.0513 (14)	0.0504 (14)	0.0146 (12)	0.0004 (12)	−0.0073 (11)
O1	0.0595 (15)	0.0880 (17)	0.0607 (14)	0.0290 (12)	−0.0135 (11)	−0.0193 (12)
C17	0.0459 (16)	0.0347 (13)	0.0336 (14)	0.0002 (11)	0.0031 (12)	−0.0001 (10)
C5	0.0438 (16)	0.0487 (17)	0.0574 (18)	0.0037 (13)	−0.0082 (14)	−0.0054 (14)
C6	0.0464 (16)	0.0359 (14)	0.0394 (14)	0.0038 (12)	−0.0018 (12)	−0.0002 (11)
C21	0.062 (2)	0.0535 (18)	0.0426 (16)	0.0045 (14)	0.0141 (14)	−0.0042 (13)
C10	0.086 (2)	0.0446 (16)	0.0396 (16)	0.0095 (16)	0.0076 (16)	−0.0013 (13)
C9	0.0574 (18)	0.0387 (14)	0.0412 (15)	0.0020 (13)	−0.0079 (13)	−0.0049 (11)
O3	0.161 (3)	0.0665 (16)	0.0589 (15)	0.0383 (16)	0.0511 (16)	0.0135 (12)
N3	0.0454 (14)	0.0581 (15)	0.0532 (15)	0.0105 (12)	−0.0046 (11)	−0.0044 (12)
C20	0.072 (2)	0.0463 (16)	0.0393 (16)	−0.0122 (15)	0.0065 (15)	−0.0061 (12)
C19	0.0537 (18)	0.0570 (18)	0.0422 (16)	−0.0130 (14)	−0.0054 (13)	0.0040 (13)
C22	0.0472 (17)	0.0555 (17)	0.0433 (16)	0.0024 (13)	0.0056 (13)	0.0001 (13)
C2	0.0432 (17)	0.0538 (18)	0.068 (2)	−0.0020 (14)	0.0022 (15)	−0.0118 (15)
C8	0.0598 (18)	0.0418 (15)	0.0401 (15)	0.0076 (13)	−0.0021 (13)	−0.0074 (12)
C1	0.0457 (17)	0.0488 (16)	0.0525 (17)	−0.0018 (13)	−0.0093 (13)	−0.0105 (13)
C4	0.062 (2)	0.0463 (16)	0.0499 (17)	−0.0020 (14)	−0.0124 (15)	−0.0095 (13)
C7	0.0543 (18)	0.0456 (16)	0.0416 (15)	0.0088 (14)	−0.0065 (13)	−0.0008 (12)
C16	0.094 (3)	0.0490 (18)	0.072 (2)	0.0104 (18)	0.027 (2)	0.0108 (16)
C11	0.071 (2)	0.0450 (16)	0.0411 (15)	0.0102 (15)	0.0085 (14)	0.0023 (12)
C13	0.091 (3)	0.0453 (19)	0.098 (3)	0.0152 (18)	0.025 (2)	0.0025 (18)
C14	0.095 (3)	0.064 (2)	0.102 (3)	0.026 (2)	0.039 (2)	0.004 (2)
C15	0.093 (3)	0.073 (3)	0.104 (3)	0.014 (2)	0.047 (2)	0.019 (2)
C12	0.069 (2)	0.0479 (17)	0.063 (2)	0.0050 (15)	0.0135 (16)	0.0039 (14)

### Geometric parameters (Å, °)

**Table:**

Br1—C3	1.899 (3)	C10—C11	1.479 (4)
Cl1—C12	1.736 (3)	C9—C8	1.531 (4)
O2—C10	1.353 (3)	C9—H9A	0.9700
O2—C8	1.441 (3)	C9—H9B	0.9700
N1—N2	1.355 (3)	C20—C19	1.361 (4)
N1—C17	1.363 (3)	C20—H20A	0.9300
N1—C9	1.452 (3)	C19—H19A	0.9300
C3—C2	1.366 (4)	C22—H22A	0.9300
C3—C4	1.369 (4)	C2—C1	1.384 (4)
C18—N3	1.382 (3)	C2—H2B	0.9300
C18—C17	1.388 (4)	C8—C7	1.532 (4)
C18—C19	1.399 (4)	C8—H8A	0.9800
N2—N3	1.301 (3)	C1—H1A	0.9300
O1—C7	1.205 (3)	C4—H4A	0.9300
C17—C22	1.395 (4)	C16—C15	1.374 (5)
C5—C4	1.374 (4)	C16—C11	1.390 (4)
C5—C6	1.385 (4)	C16—H16A	0.9300
C5—H5A	0.9300	C11—C12	1.378 (4)
C6—C1	1.388 (4)	C13—C14	1.366 (5)
C6—C7	1.492 (4)	C13—C12	1.377 (5)
C21—C22	1.365 (4)	C13—H13A	0.9300
C21—C20	1.403 (4)	C14—C15	1.371 (5)
C21—H21A	0.9300	C14—H14A	0.9300
C10—O3	1.191 (3)	C15—H15A	0.9300
			
C10—O2—C8	115.7 (2)	C21—C22—C17	115.9 (3)
N2—N1—C17	109.9 (2)	C21—C22—H22A	122.1
N2—N1—C9	120.1 (2)	C17—C22—H22A	122.1
C17—N1—C9	130.0 (2)	C3—C2—C1	120.1 (3)
C2—C3—C4	121.1 (3)	C3—C2—H2B	120.0
C2—C3—Br1	118.9 (2)	C1—C2—H2B	120.0
C4—C3—Br1	120.0 (2)	O2—C8—C9	104.9 (2)
N3—C18—C17	108.5 (2)	O2—C8—C7	109.3 (2)
N3—C18—C19	131.1 (3)	C9—C8—C7	110.4 (2)
C17—C18—C19	120.4 (3)	O2—C8—H8A	110.7
N3—N2—N1	109.3 (2)	C9—C8—H8A	110.7
N1—C17—C18	104.4 (2)	C7—C8—H8A	110.7
N1—C17—C22	133.1 (2)	C2—C1—C6	119.9 (3)
C18—C17—C22	122.5 (2)	C2—C1—H1A	120.0
C4—C5—C6	121.7 (3)	C6—C1—H1A	120.0
C4—C5—H5A	119.2	C3—C4—C5	118.8 (3)
C6—C5—H5A	119.2	C3—C4—H4A	120.6
C5—C6—C1	118.4 (2)	C5—C4—H4A	120.6
C5—C6—C7	117.6 (2)	O1—C7—C6	121.9 (3)
C1—C6—C7	124.0 (2)	O1—C7—C8	119.4 (3)
C22—C21—C20	122.4 (3)	C6—C7—C8	118.7 (2)
C22—C21—H21A	118.8	C15—C16—C11	121.8 (3)
C20—C21—H21A	118.8	C15—C16—H16A	119.1
O3—C10—O2	122.4 (3)	C11—C16—H16A	119.1
O3—C10—C11	126.5 (3)	C12—C11—C16	117.3 (3)
O2—C10—C11	111.0 (2)	C12—C11—C10	123.0 (3)
N1—C9—C8	112.4 (2)	C16—C11—C10	119.6 (3)
N1—C9—H9A	109.1	C14—C13—C12	119.9 (3)
C8—C9—H9A	109.1	C14—C13—H13A	120.1
N1—C9—H9B	109.1	C12—C13—H13A	120.1
C8—C9—H9B	109.1	C13—C14—C15	120.5 (3)
H9A—C9—H9B	107.9	C13—C14—H14A	119.8
N2—N3—C18	108.0 (2)	C15—C14—H14A	119.8
C19—C20—C21	121.5 (3)	C14—C15—C16	119.2 (4)
C19—C20—H20A	119.2	C14—C15—H15A	120.4
C21—C20—H20A	119.2	C16—C15—H15A	120.4
C20—C19—C18	117.3 (3)	C13—C12—C11	121.4 (3)
C20—C19—H19A	121.3	C13—C12—Cl1	117.0 (3)
C18—C19—H19A	121.3	C11—C12—Cl1	121.6 (2)
			
C17—N1—N2—N3	0.7 (3)	N1—C9—C8—C7	169.5 (2)
C9—N1—N2—N3	179.3 (2)	C3—C2—C1—C6	1.1 (5)
N2—N1—C17—C18	−0.3 (3)	C5—C6—C1—C2	−2.2 (4)
C9—N1—C17—C18	−178.8 (2)	C7—C6—C1—C2	179.5 (3)
N2—N1—C17—C22	−179.7 (3)	C2—C3—C4—C5	−1.3 (5)
C9—N1—C17—C22	1.8 (5)	Br1—C3—C4—C5	178.3 (2)
N3—C18—C17—N1	−0.1 (3)	C6—C5—C4—C3	0.1 (4)
C19—C18—C17—N1	−179.0 (2)	C5—C6—C7—O1	−1.2 (4)
N3—C18—C17—C22	179.4 (3)	C1—C6—C7—O1	177.1 (3)
C19—C18—C17—C22	0.4 (4)	C5—C6—C7—C8	178.2 (3)
C4—C5—C6—C1	1.6 (4)	C1—C6—C7—C8	−3.5 (4)
C4—C5—C6—C7	−180.0 (3)	O2—C8—C7—O1	−14.2 (4)
C8—O2—C10—O3	−10.7 (5)	C9—C8—C7—O1	100.7 (3)
C8—O2—C10—C11	167.3 (2)	O2—C8—C7—C6	166.4 (2)
N2—N1—C9—C8	−97.1 (3)	C9—C8—C7—C6	−78.7 (3)
C17—N1—C9—C8	81.3 (3)	C15—C16—C11—C12	0.8 (5)
N1—N2—N3—C18	−0.7 (3)	C15—C16—C11—C10	−176.8 (4)
C17—C18—N3—N2	0.5 (3)	O3—C10—C11—C12	−32.0 (6)
C19—C18—N3—N2	179.3 (3)	O2—C10—C11—C12	150.1 (3)
C22—C21—C20—C19	0.3 (5)	O3—C10—C11—C16	145.5 (4)
C21—C20—C19—C18	−0.7 (4)	O2—C10—C11—C16	−32.4 (4)
N3—C18—C19—C20	−178.4 (3)	C12—C13—C14—C15	0.0 (7)
C17—C18—C19—C20	0.3 (4)	C13—C14—C15—C16	1.3 (7)
C20—C21—C22—C17	0.4 (4)	C11—C16—C15—C14	−1.7 (6)
N1—C17—C22—C21	178.5 (3)	C14—C13—C12—C11	−0.9 (6)
C18—C17—C22—C21	−0.7 (4)	C14—C13—C12—Cl1	−179.0 (3)
C4—C3—C2—C1	0.7 (5)	C16—C11—C12—C13	0.5 (5)
Br1—C3—C2—C1	−178.9 (2)	C10—C11—C12—C13	178.0 (3)
C10—O2—C8—C9	172.5 (2)	C16—C11—C12—Cl1	178.5 (3)
C10—O2—C8—C7	−69.2 (3)	C10—C11—C12—Cl1	−3.9 (5)
N1—C9—C8—O2	−72.9 (3)		

### Hydrogen-bond geometry (Å, °)

**Table:**

*D*—H···*A*	*D*—H	H···*A*	*D*···*A*	*D*—H···*A*
C16—H16A···Cg1^i^	0.93	2.87	3.707	150
C21—H21A···O3^ii^	0.93	2.47	3.101 (4)	126

Symmetry codes: (i) *x*−1, *y*, *z*; (ii) *x*, *y*, *z*+1.
